# Androgen Deprivation therapy for Oligo-recurrent Prostate cancer in addition to radioTherapy (ADOPT): study protocol for a randomised phase III trial

**DOI:** 10.1186/s12885-022-09523-2

**Published:** 2022-05-02

**Authors:** J. Janssen, F. H. E. Staal, C. L. Brouwer, J. A. Langendijk, I. J. de Jong, R. J. A. van Moorselaar, E. Schuit, J. F. Verzijlbergen, R. J. Smeenk, S. Aluwini

**Affiliations:** 1 Department of Radiation Oncology, University Medical Center Groningen, Hanzeplein 1, Postbus 30 001, 9700 RB Groningen, the Netherlands; 2 Department of Urology, University Medical Center Groningen, Groningen, the Netherlands; 3 Department of Urology, Amsterdam University Medical Center, Amsterdam, the Netherlands; 4 Julius Center for Health Sciences and Primary Care, University Medical Center Utrecht, Utrecht University, Utrecht, the Netherlands; 5 Department of Radiology and Nuclear Medicine, Radboud University Medical Center, Nijmegen, the Netherlands; 6 Department of Radiation Oncology, Radboud University Medical Center, Nijmegen, the Netherlands

**Keywords:** Oligometastasis, Recurrence, Prostate Cancer, MDRT, SBRT, ADT, PSMA PET/CT, Phase III

## Abstract

**Background:**

More than 60% of oligo-recurrent prostate cancer (PCa) patients treated with metastasis-directed radiotherapy (MDRT) develop biochemical recurrence within 2 years. This recurrence rate emphasises the need for improved treatment and patient selection. In line with the treatment of primary PCa, the efficacy of MDRT may be enhanced when combined with androgen-deprivation therapy (ADT). Furthermore, the availability of PSMA PET/CT offers an excellent tool for optimal patient selection for MDRT. This phase III randomised controlled trial will investigate the role of the addition of ADT to MDRT in oligo-recurrent PCa patients selected with PSMA PET/CT to enhance oncological outcome.

**Methods:**

Two hundred and eighty patients will be randomised in a 1:1 ratio to the standard treatment arm (MDRT alone) or the experimental arm (MDRT + 6 months ADT). Patients with biochemical recurrence after primary treatment of PCa presenting with ≤ 4 metastases will be included. The primary endpoint is the 2.5-year metastases progression-free survival (MPFS). Secondary endpoints are acute and late toxicity, quality of life, biochemical progression-free survival, overall survival, and the sensitivity of the PSMA PET/CT for detecting oligometastases at low PSA-levels. So far, between March 2020 and December 2021, one hundred patients have been included.

**Discussion:**

This phase III randomised controlled trial will assess the possible benefit of the addition of 6 months ADT to MDRT on metastases progression-free survival, toxicity, QoL and survival in PCa patients with 1–4 recurrent oligometastatic lesions.

**Trial registration:**

ClinicalTrials.gov, NCT04302454. Registered 10 March 2020.

**Supplementary Information:**

The online version contains supplementary material available at 10.1186/s12885-022-09523-2.

## Background

After primary treatment of prostate cancer (PCa), between 27 and 53% of all patients develop biochemical recurrence (BCR) within 10 years, and this is mostly due to metastatic disease [1, 2]. The prognosis of metastatic PCa is highly heterogeneous, with the number of metastases and location being important prognostic factors for PCa-specific mortality [3]. Several studies reported a potential survival benefit for patients treated with a limited number of metastases (i.e. oligometastases) [4, 5]. Yet, according to current EAU guidelines, the standard recommended treatment for patients with distant relapses (i.e. metastases) is long-term Androgen Deprivation Therapy (ADT) [1]. Long-term systemic treatment with ADT is not a curative treatment and is associated with multiple and significant side effects having a detrimental influence on quality of life [6]. Thus, the argument has been put forward to delay long-term implementation of ADT until metastases progression [1, 7]. Hence, there is a clear need for an early stage and stratified treatment targeting oligometastases aiming at longer progression-free survival of those patients. This need has been further emphasised since the introduction of prostate specific membrane antigen (PSMA) PET/CT scan. PSMA PET/CT scans have served to improve early detection of the recurrence site(s) in cases of BCR. Early detection of recurrence has led to a remarkable increase in the number of patients diagnosed with oligometastases [8–10].

Image-guided metastases-directed radiotherapy (MDRT) holds great promise to fulfil this need for early treatment [10–12]. While level 1 evidence and guidelines are lacking, MDRT is generally accepted as first treatment option for oligometastatic PCa patients. Up till now, two published randomized phase II trials (including 62 and 54 patients) reported a benefit of MDRT over a waiting policy [13, 14]. Still, > 60% of patients treated with MDRT develop BCR within 2 years after treatment [9, 11]. The reason could be twofold, either not all recurrence sites are detected or not all sites are treated sufficiently with MDRT. To eliminate potential (micro)metastases that are not (yet) visible and to enhance the therapeutic effect, we propose to combine targeted MDRT with a short course of (neo) adjuvant ADT. This approach has already been proven successful in primary treatment of high risk PCa reducing local recurrence, improving metastases progression-free survival and overall survival [15–17]. Moreover, we propose to improve and standardize patient selection using PSMA PET/CT. To our knowledge, this is the first study investigating the role of MDRT combined with ADT in oligo-recurrent PCa patients selected with the PSMA PET/CT.

## Methods/design

### Objectives

The primary aim of this project is to test the hypothesis that the addition of ADT to MDRT in well-selected PCa patients with oligometastatic disease prolongs the metastases progression-free survival (MPFS) compared to MDRT alone.

The secondary aim is obtaining more insight into the sensitivity of the PSMA PET/CT for detecting oligometastases at low PSA-levels.

### Study design

The ADOPT-study is a multicentre open phase III randomised controlled trial. Participating centres are radiotherapy centres in the Netherlands (an updated list is available on ClinicalTrials.gov/ NCT04302454).

**Fig. 1:**
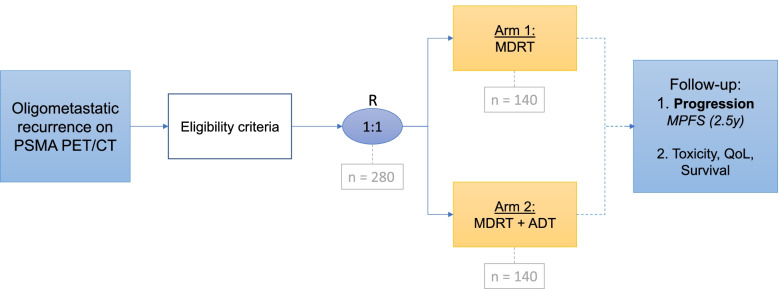
Study flowchart representing inclusion and follow-up

All eligible patients will be randomly assigned (1:1) to: MDRT alone (140 patients) versus MDRT + short term ADT (140 patients). (Fig. 1) Patients will be stratified by number of metastases, metastases organ (lymph nodes or bone, in case of both the bone group is applicable) and presence of local recurrence (identified on PSMA PET/CT).

#### Inclusion criteria

1. Histologically proven initial diagnosis of adenocarcinoma of the prostate.
2. Biochemical recurrence of prostate cancer following primary local prostate treatment (radical prostatectomy, primary radiotherapy or radical prostatectomy + prostate bed adjuvant/salvage radiotherapy).  
  BCR after surgery: PSA > 0.1 ng/ml.
  BCR after radiotherapy: PSA nadir + 2 ng/ml or 3 consequent rises in PSA level (after exclusion of possible bounce effect).
3. Minimal 1 lesion and maximum 4 lesions (bone + lymph nodes) in total on PSMA PET/CT, without evidence of visceral metastases.
  - Nodal relapse (N1) in the pelvis with a maximum of 4 positive lymph nodes. The upper limit of the pelvis is defined as the aortic bifurcation.
  - Nodal relapse (M1a) above the aortic bifurcation with a maximum of 3 positive lymph nodes.
  - Bone relapse with a maximum of 3 lesions.
  - Combination of a, b, c with a maximum of 4 metastases.
4. Age > 18 years.
5. Recent PSMA PET/CT scan within 60 days prior to randomisation.
6. PSA < 10 ng/ml.
7. In case of chronic use of finasteride or dutasteride, the PSA value should be < 5 ng/ml.
8. WHO performance state 0–2.
9. Signed informed consent prior to registration/randomisation.

#### Exclusion criteria

1. Visceral metastases.
2. PSA ≥ 10 ng/ml.
3. PSA-doubling time ≤ 3 months.
  To measure the PSA-doubling time it is advised to use an online calculating tool. Hereby it is important to include all measurable PSA values after the primary therapy (i.e., prostatectomy or (salvage)radiotherapy).
4. ADT or chemotherapy for recurrent PCa.
5. Testosterone < 1.7 nmol/l.
6. Painful metastases for which pain medication (> level 1) is indicated.
7. Invasive active cancers other than superficial non-melanoma skin cancers.
8. Inability or unwillingness to understand the information on trial-related topics, to give informed consent or to fill out QoL questionnaires.

### Primary endpoint

#### 2.5-year metastases progression-free survival (MPFS)

MPFS is defined as the time from randomisation to the date of metastases progression (appearance of new metastatic recurrence and/or progression of the treated metastases), reported by PSMA PET/CT during the follow-up (date PSMA PET/CT).

### Secondary endpoints

#### Sensitivity of the PSMA PET/CT for detecting oligometastases and pattern of recurrences after MDRT

##### Biochemical Progression-Free Survival (bPFS)

bPFS is defined as the time from the date of randomisation to the date of biochemical (PSA) recurrence. In this study a PSA-value higher than the last value prior to therapy (validated 6 weeks later) is considered a secondary biochemical recurrence (post MDRT BCR).

##### Clinical progression-free survival (PFS)

Progression is defined as biochemical progression, clinical progression (including loco-regional or distant progression on imaging) or start with hormonal therapy, whichever occurs first.

##### Acute toxicity (up to 3 months) and late toxicity (up to 3 years)

Toxicity will be assessed as reported by the patient (RTOG Acute Radiation Morbidity Criteria using adapted RTOG/EORTC acute Radiation Morbidity questionnaires) and the physician ( Common Terminology Criteria for Adverse Events (CTCAE), version 4.0).

##### Quality of life (QoL)

To evaluate QoL aspects, the European Organization for Research and Treatment of Cancer (EORTC) QoL thirty item score questionnaires (QLQ-C30) will be used, which is a cancer-specific questionnaire focusing on symptoms and side effects of specific diseases and treatments. Furthermore, the EORTC QoL prostate carcinoma module (QLQ-PR25) is used to address the specific impact of treatment-related side effects in prostate cancer.

##### Time to palliative hormonal treatment (ADT)

Time to hormonal treatment is defined as the time from trial randomisation to start of palliative hormonal treatment. Palliative ADT should not be started for biochemical progression without documented clinical progression. In case of symptomatic progression, palliative ADT is mandatory. In case of clinical asymptomatic progression, delayed ADT until progression to a symptomatic state is permitted for men who are well informed (EAU guidelines [1]).

##### Overall survival

Overall survival is defined as the time from the date of randomisation to the date of death from any cause.

*Other secondary endpoints* are disease specific survival, start of second-line treatment, start of second MDRT treatment for new (progressive) oligometastases, and the time to castration-resistance.

### Interventions

#### Radiotherapy treatment (arm 1 and arm 2)

Patients will be treated with stereotactic body radiotherapy (SBRT) to all oligometastases (up to 4). In case of > 2 pelvic lymph node metastases, whole pelvic fractionated radiotherapy (WPRT) treatment is recommended.

Patients with a post-prostatectomy local recurrence on the PSMA PET/CT with up to 4 oligometastases will be treated with SBRT to all oligometastatic lesions or WPRT and with fractionated radiotherapy to the prostate bed.

##### Patient positioning and planning CT acquisition

Patients will be positioned in supine position with indexed head, knee and feet support. Additional immobilization devices, allowing safe treatment delivery, can be used according to the institutional policy. In case of WPRT or prostate bed RT treatment with comfortably full bladder is recommended. An empty rectum is recommended for prostate bed radiotherapy.

A treatment planning CT scan is required to define the clinical target volume (CTV), the planning target volume (PTV) and the critical structures. CT slice thickness should be ≤ 2 mm. For MDRT the treatment planning CT scan should include the entire target volume and the surrounding organs at risk (OAR). For spine cases, with the aim to easily identify vertebrae, the planning CT scan should include sacrum for lower spine or include C1 for upper spine. MRI of the target vertebrae, and at least one to two vertebrae above and below, is suggested for target volume and OAR delineation. In case of WPRT or prostate bed RT, the treatment planning CT scan should include at least the pelvis from the lower part of the second lumbar vertebra (L2) to the lower part of the ischial tuberosities. If MRI is used for co-registration (recommended for prostate bed radiotherapy), the same position for both the planning CT and MRI is recommended.

##### SBRT-MDRT treatment

MDRT will be given with a SBRT technique in 3 fractionation regimens according to the location of the metastases and the surrounding organs at risk: 5 fractions of 7 Gy every other day, 3 fractions of 10 Gy (48 h interval between fractions) or 2 fractions of 12 Gy once a week. An equivalent (hypo) fractionated regimen with a EQD2 ≥ 70 Gy using alpha beta of 3 Gy is allowed.

The prescribed dose preferably covers > 95% of the PTV (V100 > 95%), and should cover at least 90% of the PTV (minor variation, V100 > 90%) (Table 1). In case of violation of dose constraints to the surrounding organs at risk, the prescription will be adapted accordingly.

**Table 1:** Three fractionation regimens and the required clinical goals for MDRT using SBRT in the ADOPT trial

Fractions (n)	Total prescribed dose (Gy)	Clinical goal	Minor variation
2	24	•PTV V100% > 95% •PTV V95% ≥ 99%	•PTV V100% > 90% •PTV V92% ≥ 99%
3	30		
5	35		

##### Whole pelvic and prostate bed treatment

The lymph node (LN) PTV will receive fractionated radiotherapy equivalent to 46–50 Gy. An integrated boost to LN > 1 cm will be given up to equivalent dose of 66 Gy if the constraints of surrounding organ at risk are not violated. An equivalent dose of 70–77 Gy should be given to prostate bed PTV synchronically with the oligometastatic lesions. Treatment plans will be regarded as optimal when > 98% of the PTV is covered by the 95% isodose and less than 2% of the PTV is higher than the 107% isodose. Treatment plans are considered as accepted with minor violation if less than 98% but more than 95% of the PTV is covered by the 95% isodose or the 90% to 94.9% isodose covers > 98% of the PTV, and if more than 2% but not more than 5% of the PTV receive a dose higher than the 107% isodose.

##### Target volumes and organs at risk (OAR)

The definition of target volumes will be in accordance with the ICRU (ICRU 62, 83, 91) report [18]. For MDRT the gross tumour volume (GTV) is the visible lesion on planning CT matched with the PSMA PET/CT. There is no GTV-CTV margin for LN advised, and for non-vertebral bone metastases a GTV-CTV margin of 5-10 mm is allowed according to local protocol. For vertebral bone metastases the use of the International Spine Radiosurgery Consortium Consensus Guideline is recommended [19]. The PTV margin advised for MDRT is GTV + 5 mm uniformal margin, but dependent on the local protocol of the treatment centre. The prostate bed and WPRT target volume recommendations are added to Additional File 1. In case of MDRT, OAR are delineated according to the location of metastases (at least up to 2 cm above the PTV). In WPRT and prostate bed RT the OAR concern bladder, rectum, bowel and femoral heads. The organ at risk constraints are added to Additional File 1.

##### Irradiation techniques and position verification

Intensity modulated radiotherapy (IMRT) or use of rotational techniques is mandatory in case of WPRT and SBRT. By definition, only dosimetry obtained by inverse treatment planning is considered as IMRT. IMRT may be performed by using Step-and-Shoot-Technique, Sliding-Window-Technique or Volumetric Modulated Arc Therapy, including MRI-guided radiation therapy systems. Daily image guidance will be used for all treatments.

#### Androgen deprivation therapy (arm 2)

The experimental arm should receive an LHRH-agonist (leuprorelin), for a duration of 6 months. The medication will be given in one administration of 45 mg (subcutaneously), the leuprorelin will be supplied on a physician prescription and administered according to the standard use of this medication for prostate cancer. Flare prevention with an anti-androgen could be used for at least 5 days prior to the first injection of the agonist and should not be continued for longer than 28 days. ADT should start no later than the first day of radiotherapy and no earlier than 12 weeks before the start of radiotherapy. Testosterone levels should be checked at 4 weeks following the first injection. ADT-related toxicity should be managed according to Nguyen et al [20].

No treatments potentially influencing the PSA level should be started before the primary endpoint is reached (metastatic progression). The total duration of ADT is limited to 6 months and is not allowed to be continued afterwards. It is highly recommended not to start ADT in case of biochemical progression without clinical progression.

### Evaluation and follow-up

At baseline the investigations include the collection of patient characteristics, a full medical history including PCa characteristics, and the results of the PSMA PET/CT imaging. Baseline toxicity (physician and patient reported) and QoL (patient reported) are recorded. Baseline laboratory assessments include PSA, Testosterone, Haemoglobin (Hb), and Alkaline phosphatase (ALP). The assessments at follow-up include changes in medical history and medication, WHO-performance score, and regular laboratory assessments. Toxicity and QoL will be regularly monitored, and adverse events are reported. The investigations required at baseline and during follow-up are summarized in Table 2.

**Table 2:** Follow up after radiotherapy treatment

Required investigation	Baseline	Treatment period		Follow-up				
			1 m after RT	3 m after RT	6 m after RT	9 m after RT	12 m after RT	Every 6 (up to 36 months) months until progression^b^
Eligibility check	X							
Informed consent	X							
ADT (arm 2)		X						
Patient characteristics/ medical history	X		X	X	X	X	X	X
Laboratory assessment								
PSA	X		X	X	X		X	X
Testosterone	X		X		X		X	X
Hb	X			X	X		X	X
ALP	X			X	X		X	X
Whole body PSMA PET/CT^a^	X						X	Every 12 months
Treatment and dosimetric data		X						
Toxicity & QoL								
CTCAE v4	X	X^c^	X	X	X		X	X
RTOG-EORTC toxicity	X	X^c^	X	X	X		X	X
EORTC-QlQ-25	X				X	X	X	X
EORTC-QLQ-C-30	X				X		X	X
SAE		X	X	X	X	X	X	X^d^

^a^ PSMA before inclusion, at progression and for patients without progression only at 12 and 24 months

^b^ 36 months visit, only lab, EORTC-QLQ-C-30 and CTCAE toxicity criteria

^c^ only in case of WPRT or prostate bed RT, the toxicity measurements should be intended at 4th week of treatment course

^d^ Until 2.5 years after treatment completion

#### Imaging

Follow-up PSMA PET/CT should be performed in case of biochemical progression and for patients without progression at 12 and 24 months after treatment. Biochemical progression is defined as a PSA-value higher than the last value prior to therapy (validated 6 weeks later).

### Safety

To ensure patient’s rights, wellbeing and safety, compliance as well as quality of data, an independent and qualified monitor will visit the sites on a regular basis. Frequency of the visits depends on the actual patient inclusion rate and the observed events and deviations.

### Statistical analysis

#### Sample size

From a previous meta-analysis, we know that the median progression-free survival in patients treated with MDRT is 21 months [6]. This corresponds to an event rate of 50% in the MDRT group at 21 months. For the MDRT + ADT group we assume a decreased event rate to 35% (65% event-free). Assuming a constant hazard of 0.0330 in the MDRT group and 0.0205 in the MDRT + ADT group, the metastases progression-free survival at 2.5 years is expected to be 37.1% in the MDRT group and 54.0% in the MDRT + ADT group. This is associated with a hazard ratio of 0.621. Assuming an alpha of 5%, power of 80%, and a dropout of 18% at 30 months, we need to recruit 140 patients per treatment arm and 280 patients in total.

#### Primary endpoint analysis

For the primary endpoint analysis, observations will be censored at 30 months. The MPFS of the two arms will be compared using a Cox proportional hazards regression model to obtain a hazard ratio and accompanying 95% confidence interval. The stratification variables will be accounted for by including these as covariates in the Cox regression [21]. Furthermore, the proportional hazard assumption will be assessed.

For subgroup analysis, an interaction analysis will be conducted to test treatment effect modification with respect to several defined factors, such as number of metastases, type of metastases and location of lymph node metastases. These subgroup analyses should be considered for hypothesis generating rather than hypothesis testing since the trial is not designed to detect these heterogeneous treatment effects.

#### Secondary endpoints analyses

Continuous outcomes will be compared using linear or quantile regression as appropriate. Categorical outcomes will be compared using logistic regression, and time-to-event endpoints will be compared using Cox regression analyses. All analyses will be performed with multivariable adjustment for stratification variables.

### Inclusion of the first 100 patients

The first patient was included March 2020 and, up to the beginning of December 2021, a total of 100 patients have been randomised at eight different Dutch treatment centres. Patients were randomised to MDRT (*n* = 49) and MDRT + ADT (*n* = 51) and were grouped to number of metastases (≤ 2 *n* = 86 |> 2 *n* = 14), bone or nodal metastases (bone *n* = 28 | nodal *n* = 72) and presence of local recurrence (present *n* = 5).

## Discussion

Current guidelines recommend long term (mostly life-long) ADT for all metastatic patients, including those with oligometastatic disease [1]. Long term ADT, however, does not provide cure, and has a detrimental effect on QoL [6]. Metastasis-directed radiotherapy can effectively ablate metastases in oligometastatic disease. However, progression is observed in > 60% of patients within 2 years after MDRT [13].

We hypothesize that the addition of short-term ADT to MDRT could eliminate undetected metastases and enhance local control of irradiated metastases. Moreover, we aim to improve and standardize patient selection using the most sensitive imaging modality at this moment (PSMA PET/CT).

Positive results of this phase III randomised controlled trial will result in longer PCa disease control, improvement in QoL, sparing of expensive second and third line treatments and potential reduction in cancer mortality.

## Supplementary Information

**Additional file 1.** Target volume definitions and dose to organs at risk.

## Data Availability

Data sharing is not applicable to this article as no datasets were generated or analysed during the current study.

## Abbreviations

PCa: Prostate cancer
BCR: Biochemical recurrence
ADT: Androgen-deprivation therapy
PSMA: Prostate specific membrane antigen
MDRT: Metastasis-directed radiotherapy
MPFS: Metastases progression-free survival
QoL: Quality of life
Hb: Haemoglobin
ALP: Alkaline phosphatase
SBRT: Stereotactic body radiotherapy
WPRT: Whole pelvic fractionated radiotherapy
CTV: Clinical target volume
PTV: Planning target volume
OAR: Organs at risk
LN: Lymph node
GTV: Gross tumour volume
IMRT: Intensity modulated radiotherapy
bPFS: Biochemical Progression-Free Survival
CTCAE: Common Terminology Criteria for Adverse Events
