# Supplementary material for: Androgen Deprivation therapy for Oligo-recurrent Prostate cancer in addition to radioTherapy (ADOPT): study protocol for a randomised phase III trial
Source: BMC Cancer. 2022 May 2;22:482. doi: 10.1186/s12885-022-09523-2 (PMC9063099; doi:10.1186/s12885-022-09523-2)
Supplement: Supplementary file 1 — Additional file 1. Target volume definitions and dose to organs at risk. [file 12885_2022_9523_MOESM1_ESM.docx]

**Additional file 1: Target volume definitions and dose to organs at risk**

**Target volumes**

The definition of target volumes will be in accordance with the ICRU (ICRU 62, 83, 91) report. (1)

**Gross tumour volume (GTV)**

SBRT MDRT: Visible lesion on planning CT (matched with the PSMA PET/CT).

**Clinical target volume (CTV)**

*SBRT MDRT:* CTV=GTV.

*Bone metastases:*

In case of non-vertebral bone metastases, a GTV-CTV margin of 5-10 mm is allowed, according to the site local protocol.

*Vertebral bone metastases:*
The use of International Spine Radiosurgery Consortium Consensus Guidelines for Target Volume Definition in Spinal Stereotactic Radiosurgery (2) is recommended.

*Prostate bed*:

Defined by any of the published consensus guidelines such as EORTC (3), RTOG (4) or ANZUP (5).

*WPRT*

The pelvic lymph node volumes to be irradiated include distal common iliac (start at L4/L5 interspace), presacral lymph nodes (S1-S3), external iliac lymph nodes, internal iliac lymph nodes, and obturator lymph nodes. Lymph node CTVs include the vessels (artery and vein) and a 7-mm radial margin being careful to ‘‘carve out’’ bowel, bladder, bone, and muscle. Volumes begin at the L4/L5 interspace and end at the superior aspect of the pubic bone.

Consensus on dose–volume histogram constraints for organ at risk (OAR) should also be attained.

**Planning target volume (PTV)**

*SBRT-MDRT*

PTV MDRT= GTV + 5 mm uniformal margins, or less according to the local protocol..

*Prostate bed and WPRT*:

PTV will provide margin around the CTV to compensate for variability in daily treatment set-up and internal CTV motion due to breathing or motion during treatment. The PTV must include the entire CTV. The CTV-PTV-margins are used according to institutional policies, but should not exceed 10 mm, nor be less than 5 mm (in case of online image guidance).

**Organs at risk (OAR)**

SBRT MDRT

All OARs (at least up to 2 cm above the PTV) are delineated in all cases according to the location of the metastases. The dose constraints to OAR for SBRT are mentioned in table 1.1. Local constraints will be applied in case of a different fractionation schedule.

**Table 1.1** Dose constraints to OAR for SBRT to metastatic lesions

| **Organ** | **Constraints** | **2x12 Gy** | **5x7 Gy** | **3x 10 Gy** |
| --- | --- | --- | --- | --- |
| Myelum | Dmax | 9 Gy/fraction=18 Gy | 5 Gy/fraction=25 Gy | 7.5 Gy/fraction=22.5 Gy |
| Esophagus | Dmax | 11Gy/fraction= 22 Gy | 7Gy/fraction=35 Gy | 9 Gy/fraction=27 Gy |
| Trachea/ larynx & main brochi | Dmax | 13 Gy/fraction=26 Gy | 9Gy/fraction=45 Gy | 10.5 Gy/fraction=31.5 Gy |
| Plexus brachialis | Any point | 10 Gy/fraction=20 Gy | 6Gy/fraction=30 Gy | 8.5 Gy/fraction=25.5 Gy |
| Liver | Any point | 700 cc must receive less than 12 Gy total | 700 cc must receive less than 17.5 Gy total | 700 cc must receive less than 15 Gy total |
| Lung | V20 (EQD2) | 11 Gy total < 30% | 16 Gy total <30% | 13.5 Gy total <30% |
| Stomach, Colon/ small bowel | D max | 11 Gy/fraction=22 Gy | 6.5 Gy/fraction = 32.5 Gy | 8.5 Gy/fraction=25.5 Gy |

WPRT / Prostate bed RT

Dose constraints to OAR for prostate bed radiotherapy and/or WPRT:

- Bladder: the bladder volume receiving a dose 70 Gy will be < 30 % and volume receiving 65 Gy < 40%.
- Rectum: V50 <50%, V60 <30%, V65 <20%, V70 < 10 %, and V75 <5 %.
- Bowel: the volume receiving 50 Gy will be < 150 ml.
- Femoral head: the volume receiving 50 Gy will be <1 % of each femoral head.

**References**

1. ICRU. International Commission on Radiation Units and Measurements Reports [Available from: <https://www.icru.org/reports/>].

2. Cox BW, Spratt DE, Lovelock M, Bilsky MH, Lis E, Ryu S, et al. International Spine Radiosurgery Consortium consensus guidelines for target volume definition in spinal stereotactic radiosurgery. Int J Radiat Oncol Biol Phys. 2012;83(5):e597-605.

3. Poortmans P, Bossi A, Vandeputte K, Bosset M, Miralbell R, Maingon P, et al. Guidelines for target volume definition in post-operative radiotherapy for prostate cancer, on behalf of the EORTC Radiation Oncology Group. Radiother Oncol. 2007;84(2):121-7.

4. Michalski JM, Lawton C, El Naqa I, Ritter M, O'Meara E, Seider MJ, et al. Development of RTOG consensus guidelines for the definition of the clinical target volume for postoperative conformal radiation therapy for prostate cancer. Int J Radiat Oncol Biol Phys. 2010;76(2):361-8.

5. Sidhom MA, Kneebone AB, Lehman M, Wiltshire KL, Millar JL, Mukherjee RK, et al. Post-prostatectomy radiation therapy: consensus guidelines of the Australian and New Zealand Radiation Oncology Genito-Urinary Group. Radiother Oncol. 2008;88(1):10-9.
